# Immunotoxicity assessment of multiwalled carbon nanotubes following whole-body inhalation exposure for 30 and 90 days in B6C3F1/N mice and 30 days in HSD:Harlan Sprague Dawley SD^®^ rats

**DOI:** 10.3389/ftox.2025.1539810

**Published:** 2025-05-26

**Authors:** Victor J. Johnson, Nigel J. Walker, Michael I. Luster, Gary R. Burleson, Michelle Cora, Gregory L. Baker, Barney Sparrow, Dori R. Germolec

**Affiliations:** ^1^ Burleson Research Technologies, Inc., Morrisville, NC, United States; ^2^ Division of Translational Toxicology, National Institute of Environmental Health Sciences, National Institutes of Health, Research Triangle Park, NC, United States; ^3^ Sarepta Therapeutics, Columbus, OH, United States; ^4^ Battelle Memorial Institute, Columbus, OH, United States

**Keywords:** multiwalled carbon nanotubes, MWCNT, immunotoxicity testing, innate immunity, humoral immunity, 1020 long multiwalled carbon nanotubes, L-MWNT-1020

## Abstract

**Background:**

Several lines of evidence suggest the possibility that inhalation exposure to multi-walled carbon nanotubes (MWCNT) at occupationally relevant doses can lead to systemic immunotoxicity. To test this hypothesis, we undertook in-depth examination of immune function in mice and rats exposed by inhalation to relatively low levels of 1020 Long Multiwalled Carbon Nanotubes (L-MWNT-1020).

**Methods:**

Studies were conducted to determine the systemic and pulmonary immunotoxic effects in mice and rats exposed to L-MWNT-1020 following whole-body inhalation for 6 h/day for 5 days/week for 30 (mice and rats) and 90 (mice) days at dose levels of 0, 0.06, 0.2, and 0.6 mg/m^3^. Additional groups were administered cyclophosphamide (CPS) as a positive control for each cohort. Following exposure, pulmonary macrophage phagocytosis, immunophenotypic analysis of immune cells populations in the spleen, and systemic immune function, including tests for humoral (T-dependent antibody response, TDAR), cell-mediated (cytotoxic T-lymphocyte [CTL] activity), and innate (Natural Killer [NK] cell activity) immunity were conducted.

**Results:**

While exposure increased pulmonary macrophage activity, no major changes were observed in any of the systemic immune parameters measured in mice exposed for 30 or 90 days. In rats, there was a slight decrease in humoral immunity coinciding with an increase in the number of splenic T cell and NK cell populations.

**Conclusion:**

Although pulmonary macrophage activity increased in mice following exposure to L-MWNT-1020, systemic immune function for the most part remained unaffected. In contrast, rats demonstrated a slight decrease in humoral immune function as well as an increase in spleen cell numbers, T cell, and NK cell populations suggesting species-specific effects on systemic immunity, however, these effects were small and their biological significance with respect to altering disease susceptibility is unclear.

## Introduction

Multi-walled carbon nanotubes (MWCNTs) are concentric tubes of rolled graphene up to 100 μm long and appear as needle-like structures. When functionalized, they have widespread industrial, mechanical engineering, and biomedical applications (NIOSH, 2013). However, in their pristine form where they tend to form agglomerates, their structure, durability, and potential for widespread exposure, particularly during manufacture, raises concerns that exposure may have significant adverse health effects. A systematic review of occupational exposure studies indicated that exposure concentrations in 85% of the studies were above the current NIOSH recommended exposure limit (REL) of 1 μg/m^3^ as an 8-h time-weighted average (TWA) (Guseva Canu et al., 2020). *In vitro* studies have shown that these pristine nanotubes induce cell necrosis/apoptosis and oxidative stress when ingested by macrophages (Orecchioni et al., 2014), while pulmonary exposure (i.e., inhalation or intratracheal instillation) in experimental animals has repeatedly caused substantial lung inflammation, fibrosis and granuloma formation (Lam et al., 2004; Warheit et al., 2004; Shvedova et al., 2005; NIOSH, 2013). Mitchell et al. (2007), conducted immunotoxicology studies in mice following whole body inhalation of MWCNTs for 7 or 14 days at dose levels of 0.3, 1 or 5 mg/m^3^. Although pulmonary inflammation was not observed, a monotonic immunosuppression was induced after 14 days of exposure as evidenced by a reduced T-dependent antibody responses (TDAR) and lymphocyte proliferation to Con A mitogen. Natural killer (NK) cell activity was also reduced but only in animals exposed at 1 mg/m^3^. Follow-up studies suggested that immunosuppression was possibly due to activation of the COX-2 pathway in the lung and subsequent release of prostaglandins (Mitchell et al., 2009). More recently, whole body inhalation to L-MWNT-1020 was shown to inhibit serum IgE levels, IL-13 production in the lung, and airway mucus production in a house dust mite model of allergic airways disease (Ihrie et al., 2019). Interestingly, in the absence of pulmonary allergy, inhalation of L-MWNT-1020 resulted in minimal changes in the lung with no indications of pulmonary inflammation (Migliaccio et al., 2021). The exacerbating effect of MWCNT on respiratory allergy was later demonstrated to be due to the house dust mite (HDM) allergen corona after adsorbing to MWCNT when combined in aqueous solution for oropharyngeal aspiration exposure (Bartone et al., 2024).

Taken together previous studies suggests that immunomodulation could represent a sensitive physiological indicator adversely affected by MWCNT exposure. To help confirm and extend previous findings we undertook in-depth studies in mice and rats to determine the immunotoxicity of L-MWNT-1020 following whole body inhalation exposure for short term to subchronic durations. Exposure levels selected for these studies were previously demonstrated not to produce significant acute toxicity in lungs of experimental animals (NTP, 2019). While these exposure levels were above the NIOSH REL of 1 μg/m^3^ for elemental carbon as a respirable mass 8-h TWA concentration, they overlapped with airborne concentrations in occupational setting where levels have been measured up to 417.91 μg/m^3^ (Dahm et al., 2018).

## Materials and methods

### Test material

A representative “long” and “thin” MWCNT was selected for testing in the Division of Translational Toxicology (DTT), NIEHS Immunotoxicology studies. The 1020 Long Multiwalled Carbon Nanotube (L-MWNT-1020) from Sun Innovations (Fremont, CA) was selected based on commercial availability of large quantities, high purity (98%), and the low amount of residual metal (nickel) catalyst (0.52% by weight). Identity and purity analyses were conducted by various analytical chemistry techniques (NTP, 2019) and the L-MWNT-1020 was stored in the original shipping containers at room temperature (approximately 25°C). Reanalysis of the lot 10031301M used for these studies showed an average length and width of 2,400 nm and 16 nm, respectively, which was in accordance with the characterization by the manufacturer as 10–30 µm length and 10–20 nm diameter. The L-MWNT-1020 was composed almost entirely of carbon and was stable and chemically unreactive; therefore, the potential pulmonary toxicity and hazard of L-MWNT-1020 should be primarily a function of physical dimensions, exposure concentration, and biopersistence.

### Study design, aerosol generation, and exposure system

**FIGURE 1:**
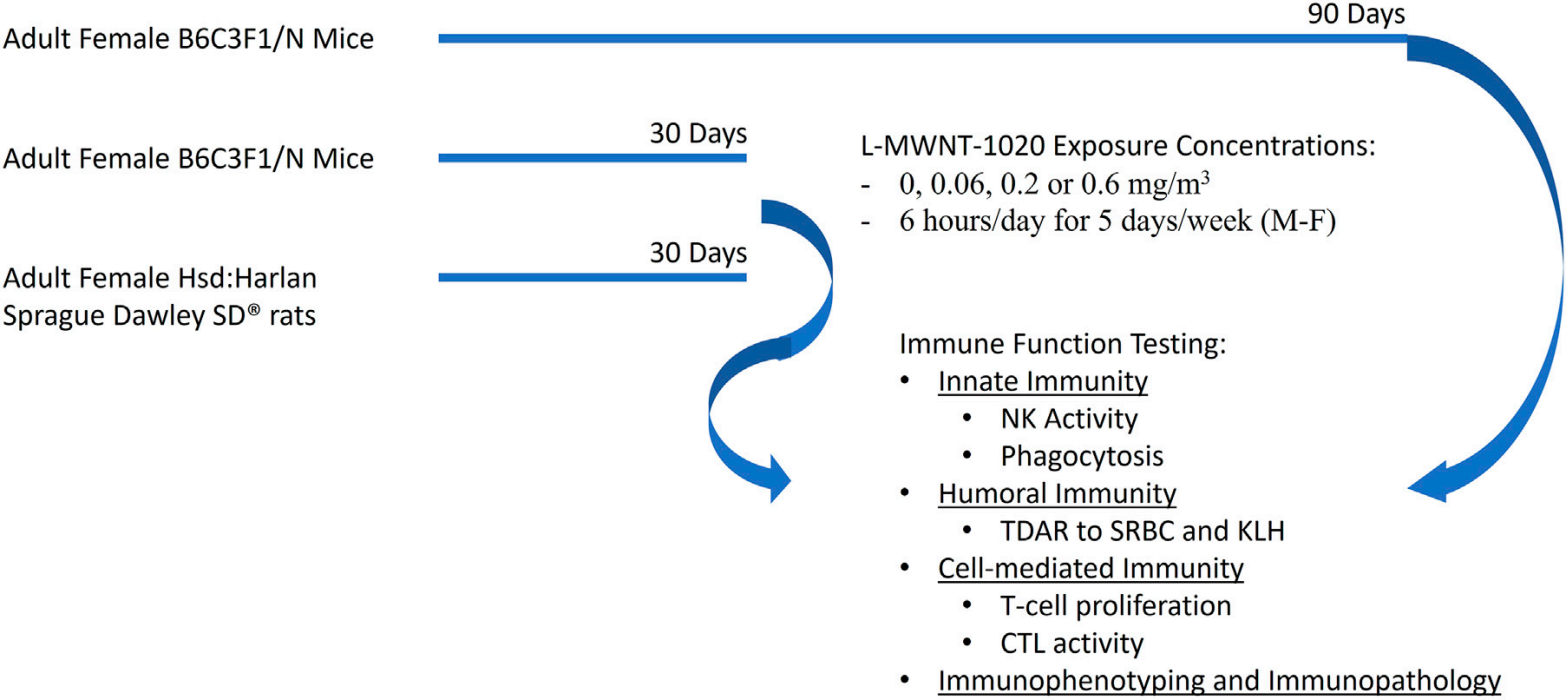
Study design for L-MWNT-1020 whole-body inhalation exposures in adult female B6C3F1/N mice and Hsd:Harlan Sprague Dawley SD^®^ rats.

All animal procedures were approved by the institutional animal care and use committee (IACUC) at the appropriate institution, Battelle Memorial Institute (Headquarters, Columbus, OH) for the inhalation exposure phase, and Burleson Research Technologies, Inc. for the immunotoxicity assessments. Exposures were conducted by Battelle Memorial Institute using whole body inhalation systems as previously described in detail (Ihrie et al., 2019; NTP, 2019). Briefly, the aerosol generation system consisted of a linear feed dust metering device to m L-MWNT-1020 from a reservoir into an air stream. A particle attrition chamber was positioned immediately downstream of the metering device exhaust tube to reduce the particle size of the test material. A single jet disperser assisted in further dispersion and particle size reduction and a cyclone separator removed the larger particles from the distribution system. Generated aerosol was conveyed from an exposure control suite to the exposure room. The inhalation exposure chamber (Lab Products, Inc., Seaford, DE) was designed so uniform aerosol concentrations could be maintained throughout the chamber with a total active mixing volume for each chamber of 1.7 m^3^. The concentrations of L-MWNT-1020 in the exposure chambers and room air were monitored using three real-time aerosol monitors. Aerosol particle size distribution and uniformity of aerosol concentration in the inhalation exposure chambers without animals present were evaluated before the studies began (NTP, 2019). Female mice and rats were used for these studies as they tend to be more sensitive to immunotoxicity and show greater dynamic range in immune responses than males (Klein and Flanagan, 2016). Female B6C3F1/N mice (Taconic Biosciences, Hudson, NY) were exposed for 30 or 90 days to L-MWNT-1020 at concentrations of 0, 0.06, 0.2 or 0.6 mg/m^3^ while cohorts of female Hsd:Harlan Sprague Dawley SD^®^ rats were exposed for 30 days at the same concentrations 6 h/day for 5 days/week, as shown in Figure 1. Following the prescribed exposure period, animals were transferred to Burleson Research Technologies, Inc. (BRT; Morrisville, NC) to provide immunizations or infections and subsequent immune function testing *ex vivo*. A positive control group was also included in which mice received 50 mg/kg bodyweight of cyclophosphamide (CPS) by intraperitoneal injection, once per day for 4–14 days (cohort dependent), prior to scheduled euthanasia and rats received 15 mg/kg bodyweight, once per day for 4–14 days (cohort dependent), prior to assessment of immune function. All animals were euthanized by CO_2_ inhalation using 100% CO_2_ introduced at 3.65 LPM into a 7.3 L chamber to displace 50% of the atmosphere per minute until breathing ceased and no pedal reflex was observed. Severing the diaphragm was used for confirmation of death.

Animals were single housed in individually ventilated cages and acclimated to the BRT vivarium for at least 7 days. Municipal tap water (Town of Cary, NC) and NTP-2000 pelleted diet were provided *ad libitum*. All animals were kept in facilities with a 12-h light/dark cycle at 19.3°C–25.0°C and with a 30%–64% relative humidity. Following acclimation, animals were randomized by bodyweight (±20% of mean bodyweight) to six independent cohorts (Table 1) for each study (30-day mouse, 90-day mouse, and 30-day rat studies). Each cohort for the mouse studies consisted of 8 animals/treatment group unless otherwise stated. Each cohort for the rat study consisted of 12 rats/treatment group with the exception of the CPS groups which consisted of 10 rats/group.

**TABLE 1:** Endpoints examined for each cohort.

Cohort	Study endpoints
1	• Clinical observations • Body and organ weights • Hematology • Pathology (enhanced immunopathology for immune tissues, traditional histology for non-immune tissues)
2	• Clinical observations • Body and organ weights • Antibody response to SRBC
3	• Clinical observations • Body and organ weights • Antibody response to KLH – In-life 5 days following immunization (IgM) and at terminal necropsy (IgG)
4	• Clinical observations • Body and organ weights • Pulmonary macrophage function • T-cell proliferation • Immunophenotyping of the spleen
5	• Clinical observations • Body and organ weights • CTL response to influenza infection
6	• Clinical observations • Body and organ weights • NK cell response to influenza infection

### Hematology (cohort 1), pathology (cohort 1), and organ weights (all cohorts)

Blood was collected by the retro-orbital (RO) route in anesthetized animals into EDTA blood collection tubes for analysis (complete hematological profiles, white blood cell differentials and reticulocyte counts). Due to unsuccessful RO blood collection, several animals had blood collected via cardiac puncture. The blood was analyzed the day of collection on an Advia 120 hematology analyzer using associated V.6.3.2-MS software (Siemens Medical Solutions United States, Inc., Malvern, PA).

Following completion of blood collection, animals were euthanized with CO_2_ and selected tissues for histopathology were collected. Gross observations were documented and the spleen, thymus, liver, right kidney with adrenals, and lungs were weighed. The spleen, thymus, mesenteric and popliteal lymph nodes, and liver (median, caudate, and right lobes) were preserved in 10% neutral buffered formalin (NBF). Other organs and tissues showing gross lesions were preserved in 10% NBF. Thymus, spleen, lymph nodes, bronchoalveolar lymphoid tissue (BALT), and bone marrow from the femur were examined using an enhanced histopathology method (Elmore, 2012), while the lung, liver, kidney (right), and adrenal gland (right) were evaluated using routine histopathology. Bone marrow cells from the right femur were removed and collected for total cell counts and cytospin preparations.

### Pulmonary mononuclear cell phagocytosis (cohort 4)

BALF was collected, and mononuclear cells (50,000 cells) were mixed with opsonized *staphylococcus aureus* bioparticles (100 μg) labeled with pH sensitive pHrodo™ Red fluorochrome (Invitrogen; Eugene, OR) that fluoresces red within phagosomes due to the acidic pH. Cytochalasin D (15 μM; Sigma, St. Louis, MO), an *in vitro* inhibitor of phagocytosis, was added to selected samples for 45 min prior to the addition of bioparticles. Cells and bioparticles were incubated for 2 h (±5 min) and then cooled to 2°C–8°C to stop phagocytosis. Phagocytosis was quantified as the increase in cell-associated red fluorescence (peak emission at 587 nm) using an Accuri C6^®^ flow cytometer using CFlow Plus^®^ software v1.0.264.15. Data were expressed as the percentage of cells that engulfed bacteria and the mean fluorescent intensity of cells that engulfed bacteria (measure of number of bacteria engulfed per cell).

### Humoral immunity (cohort 2 for SRBC and cohort 3 for KLH)

Humoral mediated immunity was assessed using two model T-dependent antigens, sheep red blood cells (SRBC, Colorado Serum Company, Denver, CO) and keyhole limpet hemocyanin (KLH; GMP-grade whole subunit, Stellar Biotechnologies, Inc., Los Angeles, CA). Four days following intravenous immunization with SRBC (7.5 × 10^7^/mouse and 1 × 10^8^/rat), the T-dependent antibody response (TDAR) was assessed by measuring the number of antibody forming cells (AFC) in the spleen using the hemolytic plaque assay, the number of IgM antibody producing B cells using ELISpot, and the serum titers of circulating IgM antibody to SRBC. A separate group received intraperitoneal immunization with KLH 14 days prior to euthanasia and serum IgM and IgG antibody titers determined 5 (IgM) and 14 (IgG) days following immunization. We have described these methods in detail previously (Watson et al., 2021).

### Splenic T-cell proliferation (cohort 4)

T-cell proliferation was examined in single cell suspensions from the spleen in response to *ex vivo* treatment with species specific monoclonal anti-CD3 antibodies (BD Bioscience, Franklin Lake, NJ), as previously described (Watson et al., 2021). Briefly, microtiter plates were coated overnight with anti-mouse CD3 antibody and then washed. Splenocytes in RPMI complete medium were added to the appropriate wells and incubated at 37°C and 5% CO_2_ for up to 96 h. A non-radioactive assay system was used to determine T-cell proliferation (CyQuant Direct Cell Proliferation Assay^®^, Molecular Probes, Eugene, OR) according to the manufacturer’s instructions. Fluorescence signal is directly proportional to DNA content and cell number. Data were collected using a SpectraMax M2e spectrofluorometer running Softmax Pro^®^ (Molecular Devices, San Jose, CA) software v5.0.1.

### Lung cytotoxic T lymphocyte (CTL) activity (cohort 5)

To stimulate an *in vivo* cell-mediated immune response, mice and rats from Cohort 5 were infected with influenza virus [∼4 × 10^4^ plaque forming units (pfu)/mouse or ∼2 × 10^5^ pfu/rat] via intranasal instillation (Burleson et al., 2018; Watson et al., 2021) 8 days prior to scheduled termination. The *ex vivo* CTL assay was performed using lung effector cells isolated from the influenza-exposed animals 8 days following infection as previously described (Watson et al., 2021). Briefly, on the day of the assay either EL-4 (mice) or UMR-106 (rat) target cells (ATCC, Manassas, VA) were infected with influenza virus (multiplicity of 10) *in vitro* and then labeled with Chromium-51 (^51^Cr at 100 μCi per 1 × 10^6^ target cells for 90 min). Lung effector cells (isolated from bronchoalveolar lavage fluid from mice or lung tissue digests from rats) were separated from red blood cells and adherent cells and combined with labeled target cells in U-bottomed microtiter plates at the appropriate effector-to-target ratios (20:1, 10:1, and 5:1 for mice or 50:1, 25:1, and 12.5:1 for rat). Plates were briefly centrifuged to facilitate effector-to-target cell contact and incubated at 37°C/5%CO2 for 6 h. Culture supernatants were harvested and release of ^51^Cr was determined using a Cobra II Auto-Gamma counter (Packard Inc., Pamsey, MN). Specific target cell lysis is a direct measure of influenza-specific CTL killing activity.

### Lung natural killer (NK) cell activity (cohort 6)

To stimulate *in vivo* recruitment and activation of NK cells in the lung, mice or rats from cohort 6 were infected with influenza virus [∼4 × 10^4^ plaque forming units (pfu)/mouse or ∼2 × 10^5^ pfu/rat] via intranasal instillation (Watson et al., 2021) 2 days prior to scheduled termination for rats and 3–4 days prior to scheduled termination for mice. Pulmonary NK cell killing activity was evaluated using YAC-1 tumor target cells as previously described (Watson et al., 2021). Briefly, lung effector cells (100 μL) were added to wells of round-bottom microtiter plates containing 100 μL of YAC-1 target cells (1 × 10^5^ cells/mL labeled with ^51^Cr at 100 μCi per 1 × 10^6^ target cells for 90 min) at effector to target ratios of 20:1, 10:1, and 5:1 for mice or 50:1, 25:1, and 12.5:1 for rats. The plates were centrifuged and then incubated at 37°C/5%CO_2_ for 4 h. Culture supernatants were harvested and release of ^51^Cr was determined using a Cobra II Auto-Gamma counter (Packard).

### Immunophenotyping of the spleen (cohort 4)

Splenic immune cell populations were determined using flow cytometry and are presented as absolute cell numbers and relative percentages of CD45^+^ lymphocytes or total myeloid cells, as appropriate. Red blood cells (RBC) present in single cell suspensions from the spleen were removed by lysis prior to Fc receptor blocking and antibody staining. Detailed procedures including antibodies, staining, and gating strategies were previously published (Watson et al., 2021). Antibody cocktails for discrimination of immune cell populations were the following.

• Rat: (1) CD45, CD3, CD45RA, and CD161a; (2) CD45, CD3, CD4, CD8a; and (3) CD11b/c, CD103, RP-1, and CD161a.
• Mouse: (1) CD45, CD3, CD45R, and CD161a; (2) CD45, CD3, CD4, CD8a; and (3) CD11b, CD11c, Ly6G, and CD335 (NKp46).

Details of the antibodies used for these studies are provided in the CEBS Supplemental Antibody Table (10.22427/NTP-DATA-500-005-001-000-1). Cell populations examined in the spleen included total lymphocytes, total T cells, CD4^+^ T cells, CD8^+^ T cells, B cells, NK cells, monocytes/macrophages, eosinophils, and neutrophils. In addition, T:B cell and CD4+:CD8+ T cell ratios were determined. Following staining, samples were stored at 2–8°C, protected from light, until analyzed on an Accuri C6 flow cytometer using CFlow Plus v 1.0.264.15 (BD Biosciences). In all cases, a minimum of 20,000 events/sample was acquired.

Lymphocyte gating was performed on CD45^+^ populations. The following lymphocyte subsets were identified; T-cells (CD3^+^CD45RA^−^), B-cells (CD3^−^CD45RA^+^), NK cells (CD3^−^CD161a^+^), T-helper cells (CD3^+^CD4^+^), and T-cytotoxic cells (CD3^+^CD8^+^). Myeloid cells were gated based on being positive for CD11b with low-to-mid intensity staining for CD11c (mouse) or CD103 (rats). Myeloid populations were differentiated from NK cells based on lack of CD335(NKp46) expression, (mouse) or lack of CD161a (rats). Further differentiation was based on expression of Ly6G (mouse) or RP-1 (rat) with positive cells being neutrophils and negative cells differentiated using SSC into monocytes/macrophages with low granularity and eosinophils with high granularity.

### Data collection, data analysis, and statistical analysis

Data were collected into Provantis v9.3.2.1 (Instem, Philadelphia, PA) and calculation of endpoints was performed within this validated electronic data collection and management system. Results are presented as mean ± SEM. Extreme values were identified by the outlier test of Dixon and Massey when sample size was <20 (Dixon and Massey, 1957) and by Tukey’s outer fences method (Tukey, 1977) when sample size was ≥20. All flagged outliers were examined by DTT, NIEHS personnel, and implausible values were eliminated from the final analyses. Jonckheere’s test was used to test for dose-related trends (Jonckheere, 1954). The positive control group was excluded from the trend tests. Bodyweight and organ weight data, which exhibit a normal distribution, were analyzed using Jonckheere’s trend test (Jonckheere, 1954) and Williams’ or Dunnett’s (pairwise) test depending on detection of a trend at a 0.01 significance level (Dunnett, 1955; Williams, 1971; Williams, 1972; Williams, 1986). Data for other endpoints were analyzed using a non-parametric multiple comparison procedure. Shirley’s test was used if a significant trend was observed (Shirley, 1977) and Dunn’s test was used if the trend was not significant (Dunn, 1964). Positive control group data were compared to the vehicle control group using the t-test for bodyweight and organ weight and Wilcoxon rank-sum test for all other endpoints. Data that were different from control at p ≤ 0.05 were considered statistically significant.

## Results

Summary findings pertinent for evaluating immunotoxicity of L-MWNT-1020 are presented below. All study findings including results that are discussed in this section but not presented in the manuscript can be found in the NTP Chemical Effects in Biological Systems (CEBS) database. (10.22427/NTP-DATA-500-005-001-000-1).

### Clinical observations, pathology, bodyweights, and organ weights (all cohorts)

In all three experimental studies occasional minor clinical abnormalities were observed, such as alopecia in mice, but were not considered related to exposure as similar incidences were observed between control and treated groups (CEBS Summary Table I05). The exposures to L-MWNT-1020 were performed by the inhalation contractor followed by shipment of the animals to BRT for assessment of immune function. L-MWNT-1020 inhalation had minimal effects on in-life bodyweights in mice and rats (CEBS Summary Tables I04 and I04G). Exposures were not continued at BRT and there were no effects on terminal bodyweights in any of the cohorts. At necropsy, the thymus, spleen, liver, right kidney with adrenals, and lungs were removed, examined, and weighed. There were no significant gross pathology (CEBS Summary Table PA46) or organ weight changes (CEBS Summary Table PA06) in any of the organs collected when compared to the vehicle control group. Histological examination revealed low numbers of pulmonary macrophages that contained black pigment, consisting of agglomerated L-MWCT-1020, in the majority of rats treated for 30 days with ≥0.2 mg/m^3^ and in all mice from the 0.6 mg/m^3^ treatment group following 90 days of exposure, but not in mice treated for 30 days. There were no other histopathological changes that could be attributed to inhalation of L-MWNT-1020 in any lymphoid or non-lymphoid tissue examined in either mice or rats (CEBS Summary Table PA02, PA03, PA08, and PA10).

### Hematology (cohort 1)

L-MWNT-1020 inhalation resulted in small, but statistically significant changes in hematological values and white blood cell (WBC) differentials. There was a significant decreasing trend with increasing exposure concentration in platelet counts in mice treated for 90 days and a significant decrease in mice treated with 0.6 mg/m^3^ L-MWNT-1020 compared to the vehicle control group. A similar negative trend in platelets was observed in rats treated for 30 days, however, no treatment groups were different from the vehicle group (CEBS Summary Table M04). Examination of WBC differentials (Table 2, CEBS Summary Table M03) indicated that there were significant increasing trends in absolute and relative neutrophils and eosinophils in rats exposed to L-MWNT-1020 for 30 days. There was also a decreasing trend in the relative lymphocyte counts in exposed rats. These trends were accompanied by significantly increased absolute neutrophil counts (0.6 mg/m^3^), relative neutrophil counts (≥0.2 mg/m^3^), and absolute and relative eosinophil counts (≥0.2 mg/m^3^), as well as significantly decreased relative lymphocyte counts (≥0.2 mg/m^3^) in rats exposed to L-MWNT-1020 for 30 days. Mice exposed to L-MWNT-1020 for 30 days appeared less sensitive and only had significantly decreased numbers of monocytes and neutrophils in the 0.06 mg/m^3^ treatment group. Extending the exposure period in mice to 90 days resulted in a significantly increasing trend in relative neutrophil counts which was significantly increased in the 0.6 mg/m^3^ group relative to the vehicle group which is consistent with the findings in rats. Mice exposed to L-MWNT-1020 for 90 days also showed significantly decreasing trends in absolute lymphocytes, basophils, and large unstained cells but only large unstained cells in mice exposed to 0.6 mg/m^3^ were significantly decreased relative to the vehicle control group (CEBS Summary Table M03). No other changes were observed in hematology parameters in mice or rats treated with L-MWNT-1020. The positive control, CPS, produced a hemogram consistent with severe leukocytopenia as expected.

**TABLE 2:** Absolute counts of immune cell populations in the spleen of rodents following inhalation of L-MWNT-1020.

	L-MWNT-1020 (mg/m^3^)				
	0	0.06	0.2	0.6	15 mg/kg CPS
30-day rat exposure study					
Total Leukocytes (x10^3^/μL)	10.104 ± 0.691 [12]	10.619 ± 0.708 [11]	9.170 ± 0.677 [11]	11.353 ± 0.516 [12]	3.050 ± 0.164 [10]**
Absolute Counts					
Lymphocytes (x10^3^/μL)	8.921 ± 0.615 [12]	9.181 ± 0.616 [11]	7.815 ± 0.543 [11]	9.633 ± 0.454 [12]	2.319 ± 0.100 [10]**
Neutrophils (x10^3^/μL)	0.664 ± 0.061 [12] ^↑↑^	0.837 ± 0.104 [11]	0.758 ± 0.080 [11]**	1.054 ± 0.092 [12]**	0.479 ± 0.043 [10]*
Monocytes (x10^3^/μL)	0.302 ± 0.027 [12]	0.358 ± 0.038 [11]	0.243 ± 0.033 [11]	0.317 ± 0.021 [12]	0.085 ± 0.008 [10]**
Eosinophils (x10^3^/μL)	0.075 ± 0.004 [12] ^↑↑^	0.100 ± 0.021 [11]	0.175 ± 0.057 [11]*	0.173 ± 0.033 [12]**	0.100 ± 0.031 [10]
Basophils (x10^3^/μL)	0.044 ± 0.006 [12]	0.041 ± 0.007 [11]	0.038 ± 0.006 [11]	0.049 ± 0.006 [12]	0.011 ± 0.002 [10]**
LUC (x10^3^/μL)	0.098 ± 0.012 [12]	0.101 ± 0.009 [11]	0.144 ± 0.042 [11]	0.126 ± 0.017 [12]	0.056 ± 0.013 [10]*
Relative Counts					
Percent Lymphocytes (%)	88.23 ± 0.48 [12] ^↓↓^	86.40 ± 0.89 [11]	85.61 ± 0.88 [11]*	84.82 ± 0.84 [12]**	76.45 ± 1.44 [10]**
Percent Neutrophils (%)	6.54 ± 0.30 [12] ^↑↑^	7.92 ± 0.72 [11]	8.17 ± 0.60 [11]*	9.27 ± 0.66 [12]**	15.63 ± 0.99 [10]**
Percent Monocytes (%)	3.09 ± 0.32 [12]	3.38 ± 0.29 [11]	2.58 ± 0.25 [11]	2.85 ± 0.21 [12]	2.77 ± 0.26 [10]
Percent Eosinophils (%)	0.78 ± 0.05 [12] ^↑↑^	0.99 ± 0.25 [11]	1.72 ± 0.46 [11]**	1.56 ± 0.29 [12]**	3.02 ± 0.76 [10]**
Percent Basophils (%)	0.41 ± 0.04 [12]	0.37 ± 0.05 [11]	0.39 ± 0.04 [11]	0.42 ± 0.04 [12]	0.36 ± 0.06 [10]
Percent LUC (%)	0.92 ± 0.06 [12]	0.95 ± 0.05 [11]	1.51 ± 0.43 [11]	1.08 ± 0.12 [12]	1.77 ± 0.37 [10]
30-day mouse exposure study					
Total Leukocytes (x10^3^/μL)	11.088 ± 0.744 [6]	8.949 ± 0.806 [8]	9.933 ± 0.468 [7]	10.009 ± 0.559 [7]	2.636 ± 0.458 [7]**
Absolute Counts					
Lymphocytes (x10^3^/μL)	9.377 ± 0.677 [6]	7.651 ± 0.716 [8]	8.403 ± 0.457 [7]	8.564 ± 0.522 [7]	2.299 ± 0.412 [7]**
Neutrophils (x10^3^/μL)	1.143 ± 0.049 [6]	0.854 ± 0.057 [8]*	1.027 ± 0.079 [7]	0.903 ± 0.059 [7]	0.220 ± 0.035 [7]**
Monocytes (x10^3^/μL)	0.162 ± 0.013 [6]	0.113 ± 0.005 [8]*	0.150 ± 0.010 [7]	0.161 ± 0.017 [7]	0.019 ± 0.005 [7]**
Eosinophils (x10^3^/μL)	0.228 ± 0.051 [6]	0.121 ± 0.014 [8]	0.174 ± 0.025 [7]	0.193 ± 0.029 [7]	0.046 ± 0.015 [7]**
Basophils (x10^3^/μL)	0.040 ± 0.010 [6]	0.045 ± 0.011 [8]	0.043 ± 0.006 [7]	0.037 ± 0.006 [7]	0.004 ± 0.002 [7]**
LUC (x10^3^/μL)	0.140 ± 0.039 [6]	0.161 ± 0.045 [8]	0.141 ± 0.023 [7]	0.146 ± 0.027 [7]	0.049 ± 0.009 [7]**
Relative Counts					
Percent Lymphocytes (%)	84.42 ± 0.72 [6]	85.26 ± 0.65 [8]	84.43 ± 0.90 [7]	85.43 ± 0.75 [7]	86.86 ± 1.46 [7]
Percent Neutrophils (%)	10.43 ± 0.49 [6]	9.88 ± 0.75 [8]	10.56 ± 1.16 [7]	9.10 ± 0.54 [7]	8.67 ± 0.74 [7]
Percent Monocytes (%)	1.50 ± 0.18 [6]	1.31 ± 0.09 [8]	1.51 ± 0.07 [7]	1.60 ± 0.13 [7]	0.70 ± 0.14 [7]*
Percent Eosinophils (%)	2.10 ± 0.51 [6]	1.36 ± 0.07 [8]	1.71 ± 0.22	2.00 ± 0.33 [7]	1.73 ± 0.52 [7]
Percent Basophils (%)	0.37 ± 0.06 [6]	0.49 ± 0.10 [8]	0.40 ± 0.06	0.39 ± 0.06 [7]	0.19 ± 0.05 [7]
Percent LUC (%)	1.18 ± 0.23 [6]	1.70 ± 0.35 [8]	1.40 ± 0.20	1.49 ± 0.30 [7]	1.89 ± 0.44 [7]
90-day mouse exposure study					
Total Leukocytes (x10^3^/μL)	9.186 ± 0.484 [8] ^↓^	9.223 ± 0.356 [6]	8.253 ± 0.450 [8]	7.523 ± 0.852 [8]	2.870 ± 0.572 [7]**
Absolute Counts					
Lymphocytes (x10^3^/μL)	7.378 ± 0.388 [8] ^↓^	7.627 ± 0.254 [6]	6.694 ± 0.359 [8]	5.954 ± 0.713 [8]	2.775 ± 0.600 [6]**
Neutrophils (x10^3^/μL)	0.969 ± 0.068 [8]	0.833 ± 0.109 [6]	0.899 ± 0.095 [8]	1.168 ± 0.176 [8]	0.202 ± 0.075 [6]**
Monocytes (x10^3^/μL)	0.084 ± 0.007 [8]	0.075 ± 0.017 [6]	0.090 ± 0.018 [8]	0.066 ± 0.027 [8]	0.000 ± 0.000 [7]**
Eosinophils (x10^3^/μL)	0.574 ± 0.055 [8]	0.503 ± 0.128 [6]	0.439 ± 0.106 [8]	0.276 ± 0.128 [8]	0.000 ± 0.000 [7]**
Basophils (x10^3^/μL)	0.061 ± 0.010 [8] ^↓^	0.062 ± 0.015 [6]	0.061 ± 0.018 [8]	0.023 ± 0.009 [8]	0.000 ± 0.000 [7]**
LUC (x10^3^/μL)	0.120 ± 0.033 [8] ^↓↓^	0.127 ± 0.036 [6]	0.068 ± 0.017 [8]	0.036 ± 0.014 [8]*	0.000 ± 0.000 [7]**
Relative Counts					
Percent Lymphocytes (%)	80.41 ± 1.08 [8]	82.95 ± 2.70 [6]	81.45 ± 2.44 [8]	78.83 ± 2.38 [8]	93.67 ± 1.09 [6]**
Percent Neutrophils (%)	10.51 ± 0.54 [8] ^↑↑^	8.97 ± 1.10 [6]	10.79 ± 0.91 [8]	16.01 ± 1.84 [8]*	6.33 ± 1.09 [6]*
Percent Monocytes (%)	0.93 ± 0.08 [8]	0.78 ± 0.17 [6]	1.06 ± 0.21 [8]	0.80 ± 0.30 [8]	0.00 ± 0.00 [7]**
Percent Eosinophils (%)	6.19 ± 0.41 [8]	5.33 ± 1.33 [6]	5.16 ± 1.30 [8]	3.51 ± 1.59 [8]	0.00 ± 0.00 [7]**
Percent Basophils (%)	0.66 ± 0.08 [8]	0.65 ± 0.15 [6]	0.71 ± 0.21 [8]	0.29 ± 0.11 [8]	0.00 ± 0.00 [7]**
Percent LUC (%)	1.34 ± 0.37 [8]	1.35 ± 0.35 [6]	0.79 ± 0.19 [8]	0.51 ± 0.21 [8]	0.00 ± 0.00 [7]**

Data are presented as Mean ± SEM [n]. SEM, Standard error; CPS, cyclophosphamide; LUC, Large Unstained Cells. Significant increasing (^↑^p < 0.05 or ^↑↑^p < 0.01) or decreasing (^↓^p < 0.05 or ^↓↓^p < 0.01) trend with increasing dose of L-MWNT-1020. Significantly different from vehicle control at *p < 0.05 of **p < 0.01.

### Phagocytosis (cohort 4)

**FIGURE 2:**
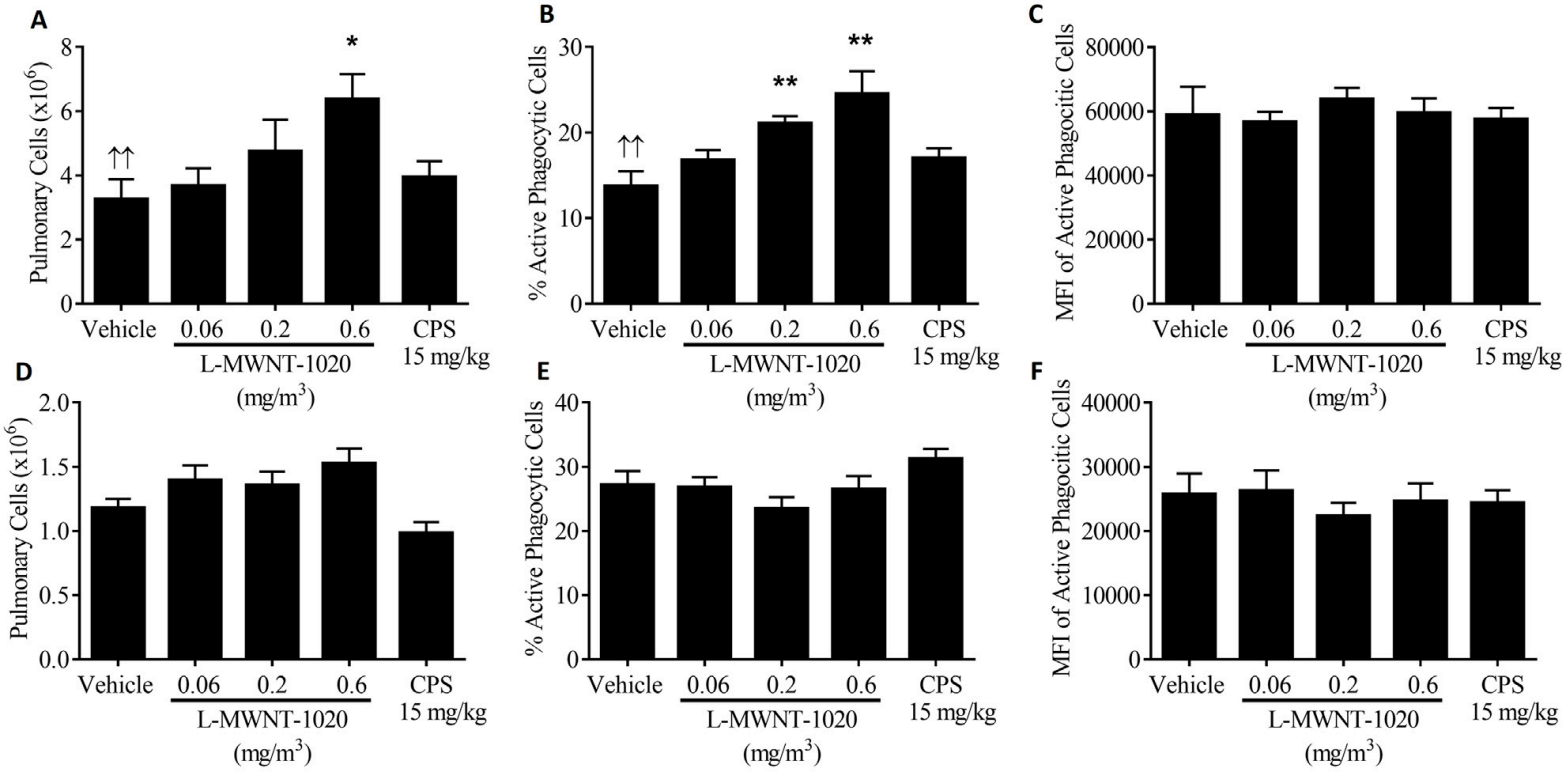
Pulmonary cell number, percent phagocytosis and mean fluorescent intensity (MFI) of phagocytized cells in mice exposed for 30 days **(A–C)**, respectively or rats exposed for 30 days **(D–F)**, respectively to 0, 0.06, 0.2 or 0.6 mg/m^3^ of L-MWNT-1020. CPS–cyclophosphamide was administered as a positive control to mice at 50 mg/kg and to rats at 15 mg/kg. SEM–standard error of the mean. Significantly different from the vehicle control group at *p < 0.05 or **p < 0.01. ^↑↑^Significant increasing dose-response trend with increasing dose of L‐MWNT‐1020 (p < 0.01).

The impact of L-MWNT-1020 exposure on the number and ability of bronchoalveolar lavage fluid (BALF) mononuclear cells to phagocytize opsonized *Staphylococcus aureus* bioparticles was determined (Figure 2; CEBS Summary Table M14). In mice exposed for 30 days, there was a significant increase in the number of pulmonary mononuclear cells present in the BALF in the 0.6 mg/m^3^ exposure group (Figure 2A) and in the percentage of mononuclear phagocytic cells actively engulfing opsonized *staphylococcus aureus* bioparticles in both the 0.2 and 0.6 mg/m^3^ exposure groups (Figure 2B). The relative number of opsonized *Staphylococcus aureus* bioparticles phagocytized per cell, as measured by mean fluorescent intensity (MFI), was not affected by exposure in mice (Figure 2C). Neither pulmonary mononuclear cell number nor phagocytosis were affected in rats exposed by inhalation for 30 days to L-MWNT-1020 (Figures 2D–F). There was insufficient uptake of the fluorescent bioparticles by the pulmonary phagocytic cells for all animals and assessment of phagocytic function was not possible in the 90-Day mouse study (data are not presented).

### Humoral immune response to T-lymphocyte dependent antigens (cohort 2 for SRBC and cohort 3 for KLH)

**FIGURE 3:**
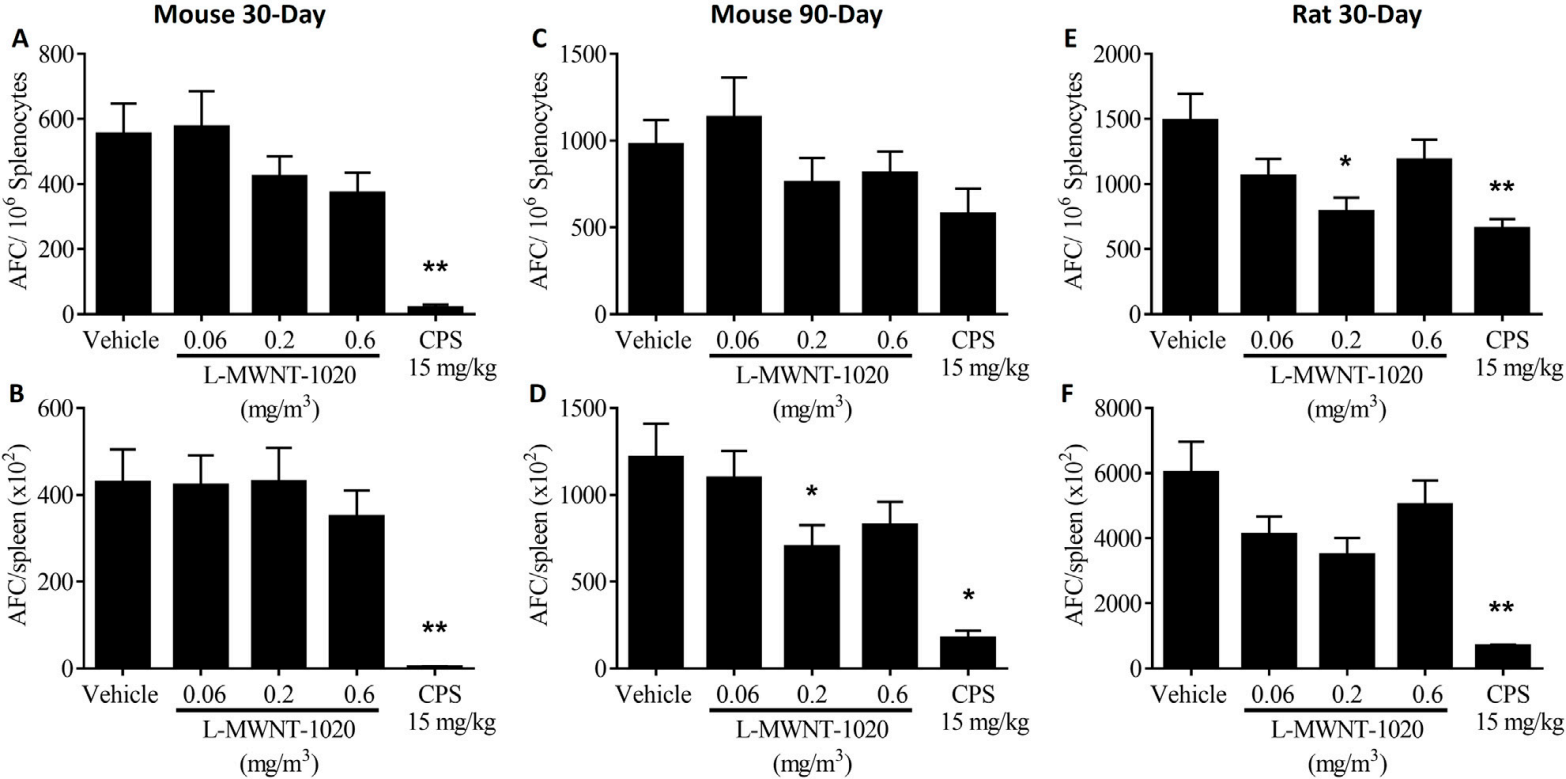
Antibody forming cell response to SRBC in rodents following inhalation of L-MWNT-1020 for 30 days in mice **(A,B)**, 90 days in mice **(C,D)** and 30 days in rats **(E,F)**. The AFC response is presented normalized per million splenocytes and as total numbers per spleen. AFC–Antibody forming cell, CPS–cyclophosphamide. Data are presented as Mean ± Standard Error (SEM). Group sizes for mice were 8 per treatment, except for vehicle group from the mouse 30 days study which was 7. Group sizes for rats were 12 per L-MWNT-1020 group and 10 per CPS group. Significantly different from the vehicle control at *p < 0.05 or **p < 0.01.

L-MWNT-1020 exposure in rats significantly decreased the anti-SRBC AFC response (CEBS Summary Table M07) at the 0.2 mg/m^3^ dose level when expressed as AFC/10^6^ spleen cells (Figure 3E). A decrease was also observed when expressed as AFC/spleen but did not reach statistical significance (Figure 3F). While the responses were also decreased at the 0.06 and 0.6 mg/m^3^ dose levels in rats, statistical significance was not achieved. Similar decreases were observed in the AFC responses in mice, but these changes did not reach statistical significance (Figures 3A–D). None of these groups showed significant dose response trends. Serum levels of anti-SRBC IgM antibodies (CEBS Summary Table M08) and anti-SRBC IgM producing B-cells (CEBS Summary Table M19) were negligibly impacted by inhalation of L-MWNT-1020 in mice and rats. In addition, antibody responses to KLH were not affected by L-MWNT-1020 inhalation in rats or mice (CEBS Summary Table M09). Humoral immune responses to SRBC and KLH were markedly decreased in all CPS treated animals as expected.

### Cell mediated immunity (cohort 4 for T-cell proliferation and cohort 5 for CTL response)

Cell mediated immune function was assessed by quantitating splenic T cell proliferation in response to stimulation with anti-CD3 monoclonal antibodies and by determining the ability of pulmonary T cells from influenza-infected animals to lyse influenza-infected target cells in a cytotoxic T lymphocyte (CTL) assay. There was a statistically significant increase in anti-CD3 antibody stimulated T cell proliferation at the lowest (0.06 mg/m^3^) dose of L-MWNT-1020 in mice exposed for 30 days but no effect was observed after 90 days in mice. A significant decrease in proliferation occurred in rats exposed to L-MWNT-1020 for 30 days, but only in the 0.2 mg/m^3^ treatment group (CEBS Summary Table M11). These changes in T-cell proliferation were minor and not dose-related. CTL activity was unaffected following exposure to L-MWNT-1020 in either mice or rats (CEBS Summary Table M12). CPS treatment markedly decreased the CTL response as expected.

### Natural Killer (NK) cell activity (cohort 6)

Natural killer (NK) cell activity was evaluated by examining the ability of pulmonary effector cells isolated 2 days following influenza infection to lyse YAC-1 tumor target cells. There was a significant decreasing trend in NK killing activity in mice exposed to L-MWNT-1020 for 90 days but only at the 5:1 E:T ratio. This trend was not observed in mice or rats exposed for 30 days. There were no other exposure-related effects on NK activity following inhalation of L-MWNT-1020 in either rats or mice (CEBS Summary Table M15).

### Immunophenotypes in the spleen (cohort 4)

The absolute number and relative percentages of immune cell populations in the spleen are presented in Tables 3, 4, respectively. Mice exposed to L-MWNT-1020 for 90 days showed a significant decreasing trend in relative percentage and absolute number of neutrophils with both measures being significantly decreased in the 0.6 mg/m^3^ treatment group. Changes in other cell populations in the spleen were mostly negligible in mice treated for 30 or 90 days. In rats exposed to 0.06 and 0.2 mg/m^3^ L-MWNT-1020 for 30 days there was a significant increase in the absolute number of spleen cells that corresponded with a significant increase in total lymphocytes in the same groups. The impact of L-MWNT-1020 on immune cell population numbers was on the T cell compartment as evidenced by significant increases in total T cells, CD4^+^ T cells, CD8^+^ T cells, and the total T cell:B cell ratios in rats treated with 0.06 and 0.2 mg/m^3^. In addition, there was a positive trend in NK cell numbers with all exposure levels being significantly elevated. CPS treatment was consistent with historical controls resulting in a marked reduction in the number of almost all cell phenotypes with B cells being the most affected and NK cells and monocytic cells the least.

**TABLE 3:** Absolute counts of immune cell populations in the spleen of rodents following inhalation of L-MWNT-1020.

	L-MWNT-1020 (mg/m^3^)				
	0	0.06	0.2	0.6	15 mg/kg CPS
30-day rat exposure study					
Spleen Cells (x10^6^)	345.95 ± 16.00 (12)	456.95 ± 28.47 (12) **	434.15 ± 21.57 (12) *	409.80 ± 19.40 (12)	165.18 ± 19.49 (10) **
Total Lymphocytes	340.954 ± 15.738 (12)	451.685 ± 28.159 (12) **	429.145 ± 21.429 (12) *	404.909 ± 19.201 (12)	162.807 ± 19.745 (10) **
Total T Cells	102.024 ± 4.983 (12)	154.826 ± 11.171 (12) **	143.832 ± 8.819 (12) **	126.047 ± 7.065 (12)	80.965 ± 9.626 (10)
CD4^+^ T Cells	57.520 ± 4.024 (12)	88.327 ± 8.269 (12) **	83.264 ± 5.535 (12) **	68.792 ± 4.107 (12)	42.012 ± 6.242 (10)
CD8^+^ T Cells	39.540 ± 2.564 (12)	60.507 ± 4.075 (12) **	54.213 ± 4.162 (12) *	51.805 ± 3.128 (12)	36.691 ± 3.383 (10)
B Cells	172.003 ± 9.675 (12)	213.994 ± 14.403 (12)	201.592 ± 11.929 (12)	194.959 ± 10.078 (12)	46.339 ± 6.996 (10) **
NK Cells	8.754 ± 0.606 (12) ^↑↑^	11.014 ± 0.762 (12) *	10.873 ± 0.371 (12) *	12.391 ± 1.061 (12) **	7.973 ± 1.259 (10)
Mono/Mac Cells	6.560 ± 0.651 (12)	6.962 ± 0.613 (12)	6.738 ± 0.269 (12)	6.655 ± 0.568 (12)	2.137 ± 0.370 (10) **
Neutrophils	5.749 ± 0.379 (12)	5.820 ± 0.325 (12)	6.982 ± 0.467 (12)	5.855 ± 0.362 (12)	4.072 ± 0.485 (10) *
Eosinophils	0.521 ± 0.046 (12)	0.488 ± 0.055 (12)	0.558 ± 0.093 (12)	0.416 ± 0.065 (12)	0.252 ± 0.032 (10) **
Total T Cells: B Cells Ratio	0.604 ± 0.028 (12)	0.727 ± 0.025 (12) **	0.716 ± 0.025 (12) *	0.650 ± 0.027 (12)	1.853 ± 0.106 (10) **
CD4^+^ T Cells: CD8^+^ T Cells Ratio	1.513 ± 0.143 (12)	1.485 ± 0.138 (12)	1.583 ± 0.102 (12)	1.339 ± 0.059 (12)	1.117 ± 0.095 (10) *
30-day mouse exposure study					
Spleen Cells (x10^6^)	78.48 ± 8.07 (8)	70.90 ± 3.65 (8)	75.20 ± 4.70 (7)	74.38 ± 3.08 (8)	19.88 ± 1.59 (8) **
Total Lymphocytes	70.403 ± 7.494 (8)	65.746 ± 3.957 (8)	70.824 ± 5.143 (7)	67.541 ± 2.900 (8)	19.357 ± 1.527 (8) **
Total T Cells	21.088 ± 2.375 (8)	20.014 ± 1.474 (8)	20.643 ± 2.052 (7)	19.558 ± 0.760 (8)	9.534 ± 0.727 (8) **
CD4^+^ T Cells	11.935 ± 1.393 (8)	11.518 ± 0.916 (8)	12.019 ± 1.216 (7)	11.036 ± 0.488 (8)	5.288 ± 0.403 (8) **
CD8^+^ T Cells	7.574 ± 0.828 (8)	7.093 ± 0.495 (8)	7.058 ± 0.760 (7)	6.999 ± 0.241 (8)	3.690 ± 0.289 (8) **
B Cells	42.438 ± 4.404 (8)	39.452 ± 2.152 (8)	43.374 ± 2.811 (7)	41.201 ± 1.966 (8)	8.284 ± 0.716 (8) **
NK Cells	3.403 ± 0.447 (8)	2.873 ± 0.193 (8)	3.550 ± 0.233 (7)	3.321 ± 0.155 (8)	0.746 ± 0.083 (8) **
Mono/Mac Cells	1.502 ± 0.175 (8)	1.339 ± 0.088 (8)	1.395 ± 0.087 (7)	1.457 ± 0.091 (8)	0.263 ± 0.023 (8) **
Neutrophils	0.067 ± 0.007 (8)	0.064 ± 0.007 (8)	0.075 ± 0.010 (7)	0.081 ± 0.009 (8)	0.008 ± 0.001 (7) **
Eosinophils	0.069 ± 0.009 (8)	0.051 ± 0.004 (8)	0.050 ± 0.005 (7)	0.066 ± 0.004 (8)	0.015 ± 0.001 (8) **
Total T Cells: B Cells Ratio	0.496 ± 0.012 (8)	0.505 ± 0.017 (8)	0.473 ± 0.031 (7)	0.477 ± 0.011 (8)	1.167 ± 0.056 (8) **
CD4^+^ T Cells: CD8^+^ T Cells Ratio	1.569 ± 0.027 (8)	1.619 ± 0.032 (8)	1.716 ± 0.044 (7) *	1.574 ± 0.023 (8)	1.434 ± 0.019 (8) **
90-day mouse exposure study					
Spleen Cells (x10^6^)	61.25 ± 4.60 (8)	58.73 ± 3.51 (8)	67.70 ± 5.47 (8)	54.63 ± 6.02 (8)	13.39 ± 0.84 (8) **
Total Lymphocytes	59.106 ± 4.258 (8)	56.090 ± 3.460 (8)	65.677 ± 5.273 (8)	53.319 ± 5.932 (8)	13.170 ± 0.802 (8) **
Total T Cells	17.811 ± 1.654 (8)	16.875 ± 1.057 (8)	20.122 ± 1.447 (8)	16.921 ± 1.722 (8)	7.336 ± 0.426 (8) **
CD4^+^ T Cells	10.101 ± 0.971 (8)	9.778 ± 0.713 (8)	11.745 ± 0.887 (8)	9.853 ± 1.103 (8)	4.222 ± 0.238 (8) **
CD8^+^ T Cells	6.332 ± 0.576 (8)	5.797 ± 0.295 (8)	6.820 ± 0.486 (8)	5.754 ± 0.544 (8)	2.717 ± 0.169 (8) **
B Cells	37.166 ± 2.338 (8)	35.475 ± 2.427 (8)	41.381 ± 3.778 (8)	32.914 ± 3.945 (8)	4.970 ± 0.473 (8) **
NK Cells	0.721 ± 0.052 (8)	0.696 ± 0.056 (8)	0.780 ± 0.085 (8)	0.562 ± 0.048 (8)	0.133 ± 0.011 (8) **
Mono/Mac Cells	1.450 ± 0.118 (8)	1.444 ± 0.092 (8)	1.538 ± 0.157 (8)	1.199 ± 0.135 (8)	0.209 ± 0.017 (8) **
Neutrophils	0.115 ± 0.013 (8) ^↓↓^	0.114 ± 0.008 (8)	0.115 ± 0.016 (8)	0.061 ± 0.007 (8) **	0.013 ± 0.002 (8) **
Eosinophils	0.098 ± 0.010 (8)	0.100 ± 0.008 (8)	0.099 ± 0.014 (8)	0.105 ± 0.012 (8)	0.032 ± 0.003 (8) **
Total T Cells: B Cells Ratio	0.475 ± 0.025 (8) ^↑^	0.480 ± 0.023 (8)	0.494 ± 0.022 (8)	0.522 ± 0.012 (8)	1.545 ± 0.141 (8) **
CD4^+^ T Cells: CD8^+^ T Cells Ratio	1.593 ± 0.041 (8)	1.678 ± 0.054 (8)	1.719 ± 0.020 (8)	1.703 ± 0.053 (8)	1.561 ± 0.036 (8)

Data are presented as Mean ± SEM (n). SEM, Standard error; CPS, cyclophosphamide; NK, Natural Killer; Mono/Mac–Monocyte/Macrophage. Significant increasing (^↑^p < 0.05 or ^↑↑^p < 0.01) or decreasing (^↓^p < 0.05 or ^↓↓^p < 0.01) trend with increasing dose of L-MWNT-1020. Significantly different from vehicle control at *p < 0.05 of **p < 0.01.

**TABLE 4:** Relative percentages of immune cell populations in the spleen of rodents following inhalation of L-MWNT-1020.

	L-MWNT-1020 (mg/m^3^)				
	0	0.06	0.2	0.6	15 mg/kg CPS
30-day rat exposure study					

Percent Total Lymphocytes	98.560 ± 0.126 (12)	98.854 ± 0.097 (12)	98.831 ± 0.080 (12)	98.801 ± 0.103 (12)	98.155 ± 0.834 (10)
Percent Total T Cells	30.126 ± 1.059 (12)	34.158 ± 0.818 (12) *	33.343 ± 0.788 (12)	31.096 ± 0.866 (12)	50.227 ± 1.259 (10) **
Percent CD4^+^ T Cells	56.102 ± 2.062 (12)	56.387 ± 1.748 (12)	57.882 ± 1.400 (12)	54.485 ± 0.943 (12)	50.467 ± 2.008 (10)
Percent CD8^+^ T Cells	38.963 ± 1.842 (12)	39.618 ± 1.623 (12)	37.588 ± 1.461 (12)	41.169 ± 0.990 (12)	46.730 ± 2.035 (10) *
Percent B Cells	50.256 ± 0.936 (12)	47.240 ± 0.657 (12)	46.847 ± 0.920 (12) *	48.114 ± 0.768 (12)	27.657 ± 1.191 (10) **
Percent NK Cells	2.558 ± 0.113 (12)	2.479 ± 0.151 (12)	2.591 ± 0.133 (12)	3.038 ± 0.200 (12)	4.806 ± 0.439 (10) **
Percent Mono/Mac Cells	1.917 ± 0.185 (12)	1.513 ± 0.087 (12)	1.589 ± 0.091 (12)	1.626 ± 0.112 (12)	1.277 ± 0.148 (10) **
Percent Neutrophils	1.666 ± 0.086 (12)	1.303 ± 0.075 (12) **	1.650 ± 0.147 (12)	1.429 ± 0.058 (12)	2.533 ± 0.259 (10) **
Percent Eosinophils	0.153 ± 0.014 (12) ^↓^	0.108 ± 0.011 (12)	0.131 ± 0.021 (12)	0.101 ± 0.016 (12) *	0.155 ± 0.015 (10)
30-day mouse exposure study					
Percent Total Lymphocytes	89.389 ± 1.672 (8)	92.449 ± 1.291 (8)	93.834 ± 1.043 (7)	90.821 ± 1.060 (8)	97.420 ± 0.335 (8) **
Percent Total T Cells	29.871 ± 0.467 (8)	30.276 ± 0.671 (8)	28.851 ± 1.282 (7)	29.014 ± 0.391 (8)	49.399 ± 1.152 (8) **
Percent CD4^+^ T Cells	56.464 ± 0.428 (8)	57.396 ± 0.496 (8)	58.153 ± 0.475 (7)	56.350 ± 0.362 (8)	55.450 ± 0.398 (8)
Percent CD8^+^ T Cells	36.025 ± 0.375 (8)	35.511 ± 0.428 (8)	33.989 ± 0.659 (7) *	35.844 ± 0.363 (8)	38.701 ± 0.262 (8) **
Percent B Cells	60.374 ± 0.561 (8)	60.198 ± 0.780 (8)	61.533 ± 1.390 (7)	60.928 ± 0.598 (8)	42.679 ± 1.038 (8) **
Percent NK Cells	4.755 ± 0.187 (8)	4.364 ± 0.100 (8)	5.039 ± 0.140 (7)	4.928 ± 0.146 (8)	3.805 ± 0.136 (8) **
Percent Mono/Mac Cells	1.902 ± 0.060 (8)	1.885 ± 0.064 (8)	1.864 ± 0.073 (7)	1.962 ± 0.095 (8)	1.323 ± 0.031 (8) **
Percent Neutrophils	0.086 ± 0.005 (8)	0.091 ± 0.011 (8)	0.104 ± 0.018 (7)	0.108 ± 0.010 (8)	0.042 ± 0.005 (7) **
Percent Eosinophils	0.088 ± 0.007 (8)	0.073 ± 0.004 (8)	0.066 ± 0.006 (7)	0.089 ± 0.004 (8)	0.074 ± 0.003 (8)
90-day mouse exposure study					
Percent Total Lymphocytes	96.654 ± 0.557 (8)	95.464 ± 0.586 (8)	97.023 ± 0.400 (8)	97.578 ± 0.367 (8)	98.446 ± 0.208 (8) **
Percent Total Lymphocytes	96.654 ± 0.557 (8)	95.464 ± 0.586 (8)	97.023 ± 0.400 (8)	97.578 ± 0.367 (8)	98.446 ± 0.208 (8) **
Percent Total T Cells	29.830 ± 1.069 (8) ^↑^	30.144 ± 0.968 (8)	30.854 ± 0.884 (8)	31.973 ± 0.468 (8)	55.973 ± 1.955 (8) **
Percent CD4^+^ T Cells	56.593 ± 0.643 (8)	57.716 ± 0.733 (8)	58.279 ± 0.458 (8)	57.925 ± 0.763 (8)	57.623 ± 0.543 (8)
Percent CD8^+^ T Cells	35.618 ± 0.542 (8)	34.554 ± 0.713 (8)	33.911 ± 0.256 (8)	34.164 ± 0.666 (8)	36.988 ± 0.531 (8)
Percent B Cells	63.281 ± 1.224 (8)	63.164 ± 0.971 (8)	62.723 ± 1.075 (8)	61.381 ± 0.556 (8)	37.490 ± 1.973 (8) **
Percent NK Cells	1.226 ± 0.046 (8)	1.235 ± 0.047 (8)	1.205 ± 0.102 (8)	1.083 ± 0.069 (8)	1.004 ± 0.036 (8) **
Percent Mono/Mac Cells	2.379 ± 0.111 (8)	2.467 ± 0.093 (8)	2.251 ± 0.096 (8)	2.198 ± 0.045 (8)	1.563 ± 0.076 (8) **
Percent Neutrophils	0.185 ± 0.013 (8) ^↓↓^	0.193 ± 0.005 (8)	0.165 ± 0.013 (8)	0.118 ± 0.014 (8) **	0.095 ± 0.010 (8) **
Percent Eosinophils	0.161 ± 0.011 (8)	0.170 ± 0.008 (8)	0.143 ± 0.011 (8)	0.192 ± 0.011 (8)	0.244 ± 0.017 (8) **
Percent Mono/Mac Cells	2.379 ± 0.111 (8)	2.467 ± 0.093 (8)	2.251 ± 0.096 (8)	2.198 ± 0.045 (8)	1.563 ± 0.076 (8) **
Percent Neutrophils	0.185 ± 0.013 (8) ^↓↓^	0.193 ± 0.005 (8)	0.165 ± 0.013 (8)	0.118 ± 0.014 (8) **	0.095 ± 0.010 (8) **
Percent Eosinophils	0.161 ± 0.011 (8)	0.170 ± 0.008 (8)	0.143 ± 0.011 (8)	0.192 ± 0.011 (8)	0.244 ± 0.017 (8) **

Data are presented as Mean ± SEM (n). SEM – Standard error, CPS – cyclophosphamide; NK – Natural Killer; Mono/Mac – Monocyte/Macrophage. Significant increasing (^↑^p < 0.05 or ^↑↑^p < 0.01) or decreasing (^↓^p < 0.05 or ^↓↓^p < 0.01) trend with increasing dose of L-MWNT-1020. Significantly different from vehicle control at *p < 0.05 of **p < 0.01.

## Discussion

In depth immunotoxicity studies were conducted in mice exposed for 30 and 90 days and in rats for 30 days to whole body inhalation of L-MWNT-1020 at concentrations of 0.06, 0.2, and 0.6 mg/m^3^ 6 h/day for 5 days/week. The low and mid exposure levels used in the present studies were within the range of concentrations (<0.001–417.91 μg/m^3^) previously observed in US facilities manufacturing carbon nanotubes and nanofibers (Dahm et al., 2018). Critical effect levels have already been estimated for noncancerous lung effects from animal dose response data (e.g., BMD, benchmark dose and BMDL, the 95% lower confidence limit estimates of the BMD) and extrapolated to humans for MWCNTs by accounting for the factors influencing the lung dose in experimental animals. Based on BMD modeling of subchronic animal inhalation studies (Ma-Hock et al., 2009; Pauluhn, 2010), a working lifetime exposure of 0.2–2 µg/m3 (8-h TWA concentration) was estimated to be associated with a 10% excess risk of early-stage adverse lung effects (95% lower confidence limit estimates). In view of these health risks, and ongoing improvements in sampling and analytical methodologies, NIOSH has recommended a REL of 1 μg/m^3^ as an 8-h TWA respirable mass concentration using NIOSH Method 5040 (https://www.cdc.gov/niosh/docs/2013-145/pdfs/2013-145.pdf ). The NIOSH REL is based on minimizing the risk of pulmonary inflammation, including neutrophil influx, and pulmonary fibrosis that were observed in animal studies following short-term and subchronic exposure to carbon nanotubes and nanofibers (Bergamaschi et al., 2021). Although there was systemic neutrophilia in the blood of rats exposed for 30 days to L-MWNT-1020, there was no histopathological evidence of inflammation and neutrophil influx into the lungs of rats or mice in these studies.

Experimental animal studies have established that the lung is the primary target following inhalation of single walled carbon nanotubes (SWCNTs) characterized by persistent pulmonary fibrosis, granulomas and other tissue injury (Lam et al., 2004; Shvedova et al., 2005). These studies were normally conducted at higher concentrations (e.g., 1–5 mg/kg bodyweight) than employed in the present studies and the materials were often administered as a bolus, i.e., instilled or aspirated. It is known that there can be significant differences in toxicity due to exposure method (e.g., inhalation vs aspiration or instillation) as well as between different lots of carbon nanotubes due to the length, presence of contaminants such as metal oxides, and the degree of agglomeration (Pauluhn, 2010; Vitkina et al., 2016; Fujita et al., 2020). Ninety-day studies in rats showed a moderate level of pathology at dose levels higher than 0.4–0.5 mg/m^3^ in the upper respiratory tract, including goblet cell hyper- and/or metaplasia, eosinophilic globules, and focal turbinate remodeling, but not at lower doses (Ma-Hock et al., 2009; Pauluhn, 2010). The exposures in the current studies were intentionally selected to not induce remarkable lung pathology. Previous studies conducted by the NTP using whole body inhalation exposure of L-MWNT-1020 in rats and mice indicated that chronic inflammation and alveolar (rats) or bronchiolar (mice) epithelial hyperplasia occurred only following exposure to ≥1 mg/m^3^. The chronic inflammation was characterized by diffusely scattered macrophages in alveoli as well as aggregates of cellular debris and neutrophils near alveolar ducts and terminal bronchioles indicating that the inflammation was associated mainly with the distal airways. The increased incidence of foreign body inclusions in the mediastinal and bronchial lymph nodes was mainly associated with macrophages in paracortex and medullary cords and showed increasing severity with increasing exposure concentration (NTP, 2019).

Despite the absence of lung tissue pathology, increased numbers of pulmonary macrophage and phagocytic capabilities were observed in mice exposed to L-MWNT-1020 for 30 days. This effect was not observed in rats. Shvedova et al. (Shvedova et al., 2008) has reported increased numbers of activated alveolar macrophages in the lungs of mice following a single oropharyngeal aspiration administration of single walled carbon nanotubes (SWCNTs) at doses as low as 10 μg/mouse. More importantly, they showed that pulmonary clearance of administered *Listeria* monocytogenes decreased despite the presence of these macrophages hypothesizing that the macrophages were already activated by the SWCNT exposure and were deficient in their antibacterial activities. This is consistent with *in vitro* studies investigating the impact of long MWCNTs on human primary macrophages. For up to 5 days following *in vitro* exposure, superoxide and inflammatory mediator release were increased. However, these cells had poor phagocytic capabilities, characterized by incomplete engulfment of the MWCNTs, which interfered with bacterial phagocytosis, a state known as frustrated phagocytosis (Sweeney et al., 2015). Since the phagocytosis assay performed in the present study utilized killed bioparticles, it was not possible to determine if the bactericidal activity of pulmonary macrophages was negatively impacted by inhalation of L-MWNT-1020.

Systemic effects on immune cell populations in the peripheral blood were observed in the present study. Interestingly, the effects on blood cells showed species and time differences in sensitivity. Rats exposed to L-MWNT-1020 for 30 days showed evidence of systemic inflammation with increased neutrophils and eosinophils in the blood. In contrast, mice exposed for 30 days did not show any changes in peripheral blood leukocytes. Only after 90 days of exposure did mice show some changes, mainly a small increase in the percentage of neutrophils but not absolute counts. These data suggest that rats may be more sensitive to systemic inflammation following carbon nanotube inhalation. For the most part, systemic immune functions in mice were unaffected by exposure to L-MWNT-1020 including antibody-mediated immunity, NK cell function, T cell proliferation, CTL activity, and immunophenotypes in the spleen. Earlier studies conducted by Mitchell et al. (2007), demonstrated that a monotonic decrease in the AFC response occurred in mice exposed by inhalation to 0.3, 1 or 3 mg/m^3^ MWCNT for 14 days as well as decreases in NK cell activity. Although we did not confirm these findings in mice, we did observe a slight decrease in the AFC response in rats evidenced by a significant decrease only in the 0.2 mg/m^3^ treatment group, thus the effect was not dose responsive. The lack of an effect at the high dose may be due to different mechanisms of toxicity operating at higher doses due to a higher burden and reduced clearance of L-MWNT-1020. In fact, the 0.6 mg/m^3^ dose level may be approaching lung overload in the rat as previous studies have demonstrated moderate lung overload at doses as low as 0.4 mg/m^3^ of Baytubes^®^ MWCNTs (Pauluhn, 2010) and clear evidence of lung overload in rats exposed to ≥3 mg/m^3^ L-MWNT-1020 (NTP, 2019). Consistent with the low dose effect on AFC, rats exposed to 0.06 and 0.2 mg/m^3^ showed significant changes in the constitution of immune cell populations in the spleen that were not apparent in the high dose group. These phenotypic changes included increases in total spleen cells accompanied by an increase in T-cell populations, both CD4 and CD8, and NK cells. These effects were more prominent at the lower exposure levels, further suggesting possible alterations in the exposure characteristics and/or delivery to and deposition in the airways, as the concentration MWCNTs increased. Systemic intravenous administration of PEGylated MWCNT was shown to suppress the TDAR following immunization with SRBC (Zhang et al., 2017). Although systemic distribution of the inhaled L-MWNT-1020 was not measured in the present study, it is possible that systemic absorption following inhalation exposure was responsible for the systemic effects on the immune system.

In conclusion, inhalation exposure to L-MWNT-1020 for 30 or 90 days in female B6C3F1/N mice and 30 days in female Harlan Sprague Dawley (HSD) rats at concentrations of 0, 0.06, 0.2 or 0.6 mg/m^3^ produced minimal evidence of systemic immunotoxicity. While exposure increased pulmonary macrophage activity in mice, as well as systemic inflammation evidenced by increased neutrophils in rats and mice, no significant changes were observed in mice in any of the systemic functional immune parameters measured. However, in rats there was a slight decrease in humoral immunity coinciding with an increase in splenic T cell and NK cell populations. The biological significance of the minor systemic effects observed in rats at exposure levels similar to those observed in the workplace is unclear as there was a lack of pulmonary inflammation.

## Data Availability

The datasets presented in this study can be found in online repositories. The names of the repository/repositories and accession number(s) can be found below: NIEHS CEBS (10.22427/NTP-DATA-500-005-001-000-1).

## References

[B1] Bartone R. D. Tisch L. J. Dominguez J. Payne C. K. Bonner J. C. (2024). House dust mite proteins adsorb on multiwalled carbon nanotubes forming an allergen corona that intensifies allergic lung disease in mice. ACS Nano 18, 26215–26232. 10.1021/acsnano.4c07893 39259863 PMC11440643

[B2] Bergamaschi E. Garzaro G. Jones G. W. Buglisi M. Caniglia M. Godono A. (2021). Occupational exposure to carbon nanotubes and carbon nanofibres: more than a cobweb. Nanomaterials 11 (3), 1–15. 10.3390/nano11030745 PMC8002294 33809629

[B3] Burleson G. R. Burleson F. G. Dietert R. R. (2018). Evaluation of cell-mediated immune function using the cytotoxic T-lymphocyte assay. Methods Mol. Biol. 1803, 199–208. 10.1007/978-1-4939-8549-4_13 29882141

[B4] Dahm M. M. Schubauer-Berigan M. K. Evans D. E. Birch M. E. Bertke S. Beard J. D. (2018). Exposure assessments for a cross-sectional epidemiologic study of US carbon nanotube and nanofiber workers. Int. J. Hyg. Environ. Health 221 (3), 429–440. 10.1016/J.IJHEH.2018.01.006 29339022

[B5] Dixon W. J. Massey F. J. (1957). Introduction to statistical analysis. 2nd ed. New York, NY: McGraw-Hill Book Company, Inc. (International student edition).

[B6] Dunn O. J. (1964). Multiple comparisons using rank sums. Technometrics 6 (3), 241–252. 10.1080/00401706.1964.10490181

[B7] Dunnett C. W. (1955). A multiple comparison procedure for comparing several treatments with a control. J. Am. Stat. Assoc. 50, 1096–1121. 10.2307/2281208

[B8] Elmore S. A. (2012). Enhanced histopathology of the immune system: a review and update. Toxicol. Pathol. 40 (2), 148–156. 10.1177/0192623311427571 22089843 PMC3465566

[B9] Fujita K. Obara S. Maru J. Endoh S. (2020). Cytotoxicity profiles of multi-walled carbon nanotubes with different physico-chemical properties. Toxicol. Mech. Methods 30 (7), 477–489. 10.1080/15376516.2020.1761920 32345130

[B10] Guseva Canu I. Batsungnoen K. Maynard A. Hopf N. B. (2020). State of knowledge on the occupational exposure to carbon nanotubes. Int. J. Hyg. Environ. Health 225, 113472. 10.1016/j.ijheh.2020.113472 32035287

[B11] Ihrie M. D. Taylor-Just A. J. Walker N. J. Stout M. D. Gupta A. Richey J. S. (2019). Inhalation exposure to multi-walled carbon nanotubes alters the pulmonary allergic response of mice to house dust mite allergen. Inhal. Toxicol. 31 (5), 192–202. 10.1080/08958378.2019.1643955 31345048 PMC6697090

[B12] Jonckheere A. R. (1954). A distribution free k-sample test against ordered alternatives. Biometrika 41 (1–2), 133–145. 10.1093/BIOMET/41.1-2.133

[B13] Klein S. L. Flanagan K. L. (2016). Sex differences in immune responses. Nat. Rev. Immunol. 16 (10), 626–638. 10.1038/nri.2016.90 27546235

[B14] Lam C. W. James J. T. McCluskey R. Hunter R. L. (2004). Pulmonary toxicity of single-wall carbon nanotubes in mice 7 and 90 days after intratracheal instillation. Toxicol. Sci. 77 (1), 126–134. 10.1093/TOXSCI/KFG243 14514958

[B15] Ma-Hock L. Treumann S. Strauss V. Brill S. Luizi F. Mertler M. (2009). Inhalation toxicity of multiwall carbon nanotubes in rats exposed for 3 months. Toxicol. Sci. 112 (2), 468–481. 10.1093/TOXSCI/KFP146 19584127

[B16] Migliaccio C. T. Hamilton R. F. Shaw P. K. Rhoderick J. F. Deb S. Bhargava R. (2021). Respiratory and systemic impacts following MWCNT inhalation in B6C3F1/N mice. Part Fibre Toxicol. 18 (1), 16. 10.1186/S12989-021-00408-Z 33771183 PMC7995731

[B17] Mitchell L. A. Gao J. Wal R. Gigliotti A. Burchiel S. W. Mcdonald J. D. (2007). Pulmonary and systemic immune response to inhaled multiwalled carbon nanotubes. Toxicol. Sci. 100 (1), 203–214. 10.1093/toxsci/kfm196 17660506

[B18] Mitchell L. A. Lauer F. T. Burchiel S. W. McDonald J. D. (2009). Mechanisms for how inhaled multiwalled carbon nanotubes suppress systemic immune function in mice. Nat. Nanotechnol. 4 (7), 451–456. 10.1038/nnano.2009.151 19581899 PMC3641180

[B19] NIOSH (2013). Current intelligence bulletin 65: occupational exposure to carbon nanotubes and nanofibers. Cincinnati, OH: DHHS (NIOSH) Publication, 2013–2145.

[B20] NTP (2019). Toxicity studies of 1020 long multiwalled carbon nanotubes (L-MWNT-1020) administered by inhalation to Sprague Dawley (Hsd:Sprague Dawley SD) rats and B6C3F1/N mice. Toxic. Rep. Ser. (94). 10.22427/NTP-TOX-94 PMC8040349 33529178

[B21] Orecchioni M. Bedognetti D. Sgarrella F. Marincola F. M. Bianco A. Delogu L. G. (2014). Impact of carbon nanotubes and graphene on immune cells. J. Transl. Med. 12 (1), 138. 10.1186/1479-5876-12-138 24885781 PMC4067374

[B22] Pauluhn J. (2010). Subchronic 13-week inhalation exposure of rats to multiwalled carbon nanotubes: toxic effects are determined by density of agglomerate structures, not fibrillar structures. Toxicol. Sci. 113 (1), 226–242. 10.1093/TOXSCI/KFP247 19822600

[B23] Shirley E. (1977). A non-parametric equivalent of Williams’ test for contrasting increasing dose levels of a treatment. Biometrics 33 (2), 386–389. 10.2307/2529789 884197

[B24] Shvedova A. A. Fabisiak J. P. Kisin E. R. Murray A. R. Roberts J. R. Tyurina Y. Y. (2008). Sequential exposure to carbon nanotubes and bacteria enhances pulmonary inflammation and infectivity. Am. J. Respir. Cell. Mol. Biol. 38 (5), 579–590. 10.1165/RCMB.2007-0255OC 18096873 PMC2335338

[B25] Shvedova A. A. Kisin E. R. Mercer R. Murray A. R. Johnson V. J. Potapovich A. I. (2005). Unusual inflammatory and fibrogenic pulmonary responses to single-walled carbon nanotubes in mice. Am. J. Physiol. Lung Cell. Mol. Physiol. 289 (5 33-5), L698–L708. 10.1152/ajplung.00084.2005 15951334

[B26] Sweeney S. Grandolfo D. Ruenraroengsak P. Tetley T. D. (2015). Functional consequences for primary human alveolar macrophages following treatment with long, but not short, multiwalled carbon nanotubes. Int. J. Nanomedicine 10, 3115–3129. 10.2147/IJN.S77867 25960651 PMC4412488

[B27] Tukey J. W. (1977). “Exploratory data analysis,” in The concise encyclopedia of statistics (New York, NY: Springer), 61–100. 10.1007/978-0-387-32833-1_136

[B28] Vitkina T. I. Yankova V. I. Gvozdenko T. A. Kuznetsov V. L. Krasnikov D. V. Nazarenko A. V. (2016). The impact of multi-walled carbon nanotubes with different amount of metallic impurities on immunometabolic parameters in healthy volunteers. Food Chem. Toxicol. 87, 138–147. 10.1016/J.FCT.2015.11.023 26683310

[B29] Warheit D. B. Laurence B. R. Reed K. L. Roach D. H. Reynolds G. A. M. Webb T. R. (2004). Comparative pulmonary toxicity assessment of single-wall carbon nanotubes in rats. Toxicol. Sci. 77 (1), 117–125. 10.1093/TOXSCI/KFG228 14514968

[B30] Watson ALTD Johnson V. J. Luster M. I. Burleson G. R. Fallacara D. M. Sparrow B. R. (2021). Immunotoxicity studies of sulfolane following developmental exposure in Hsd:Sprague Dawley SD rats and adult exposure in B6C3F1/N mice. J. Immunotoxicol. 18 (1), 1–12. 10.1080/1547691X.2020.1869355 34357831 PMC8462997

[B31] Williams D. A. (1971). A test for differences between treatment means when several dose levels are compared with a zero dose control. Biometrics 27 (1), 103–117. 10.2307/2528930 5547548

[B32] Williams D. A. (1972). The comparison of several dose levels with a zero dose control. Biometrics 28 (2), 519–531. 10.2307/2556164 5037867

[B33] Williams D. A. (1986). A note on Shirley’s nonparametric test for comparing several dose levels with a zero-dose control. Biometrics 42 (1), 183–186. 10.2307/2531254 3719054

[B34] Zhang T. Tang M. Zhang S. Hu Y. Li H. Zhang T. (2017). Systemic and immunotoxicity of pristine and PEGylated multi-walled carbon nanotubes in an intravenous 28 days repeated dose toxicity study. Int. J. Nanomedicine 12, 1539–1554. 10.2147/IJN.S123345 28280324 PMC5339008

